# The Race Structure of the Rice Blast Pathogen Across Southern and Northeastern China

**DOI:** 10.1186/s12284-017-0185-y

**Published:** 2017-10-05

**Authors:** Yaling Zhang, Qiongle Zhu, Yongxiang Yao, Zhenghong Zhao, James C. Correll, Ling Wang, Qinghua Pan

**Affiliations:** 1 State Key laboratory for Conservation and Utilization of Subtropical Agro-bioresources, Guangdong Provincial Key Laboratory for Crop Molecular Breeding, College of Agriculture, South China Agricultural University, Guangzhou, 510642 China; 2 College of Agronomy, Heilongjiang Bayi Agricultural University, Daqing, 163319 China; 3 Dandong Academy of Agricultural Sciences, Dandong, 118109 China; 4 Hunan Rice Research Institute, Changsha, 410125 China; 5 Department of Plant Pathology, University of Arkansas, Fayetteville, AR 72701 USA

**Keywords:** *Oryza sativa*, *Magnaporthe oryzae*, Differential cultivar, Race structure, Resistance gene

## Abstract

**Background:**

Rice blast, caused by the ascomycete *Magnaporthe oryzae* (*Mo*), imposes a major constraint on rice productivity. Managing the disease through the deployment of host resistance requires a close understanding of race structure of the pathogen population.

**Results:**

The host/pathogen interaction between isolates sampled from four *Mo* populations collected across the rice-producing regions of China was tested using two established panels of differential cultivars. The clearest picture was obtained from the Chinese cultivar panel, for which the frequency of the various races, the race diversity index, the specific race isolate frequency, and the frequency of the three predominant races gave a consistent result, from which it was concluded that the pathogen population present in the southern production region was more diverse than that in the northeastern region. The four blast resistance genes *Pi1*, *Pik*, *Pik-m*, and *Piz* all still remain effective in the southern China rice production area, as does *Pi1* in the northeastern region. The effectiveness of *Pita*, *Pik-p*, *Piz*, and *Pib* is restricted to single provinces. The distinctive resistance profile shown by the Chinese differential cultivar set implied the presence of at least five as yet unidentified blast resistance genes.

**Conclusions:**

The Chinese differential cultivar set proved to be more informative than the Japanese one for characterizing the race structure of the rice blast pathogen in China. A number of well characterized host resistance genes, in addition to some as yet uncharacterized ones, remain effective across the major rice production regions in China.

**Electronic supplementary material:**

The online version of this article (doi: 10.1186/s12284-017-0185-y) contains supplementary material, which is available to authorized users.

## Background

Rice blast, caused by the heterothallic ascomycete *Magnaporthe oryzae* (*Mo*) represents one of the most serious biotic constraints over the yield of rice (Couch and Kohn 2002; Singh et al. 2015; Liang et al. 2016; Deng et al. 2017). Its management relies heavily on the deployment of host resistance genes (He et al. 2012; Zhai et al. 2014; Deng et al. 2017). Host resistance largely follows the classical gene-for-gene relationship in which a host gene specifies resistance and a matching pathogen gene specifies avirulence (Flor 1971; Burdon and Thrall 2009). Major resistance genes are prone to rapid breakdown as a result of the high genetic instability and diversity of the pathogen or the ambiguous characteristics about race (also called as physiological race, or pathotype) structures in the targeted regions (Zeigler et al. 1995; Mekwatanakarn et al. 2000; Wu et al. 2014; Zhang et al. 2015; Wang et al. 2017). Characterizing both the pathogen population race structure and the host resistance gene content are key to formulating a viable deployment strategy of host resistance in any given production region (Xia et al. 2000; Wu et al. 2014; Zhang et al. 2015; Kawasaki-Tanaka et al. 2016; Wang et al. 2017).

An isolate’s race is conventionally defined by its profile of pathogenicity to a panel of cultivars chosen to harbor a spectrum of known resistance genes (Atkins et al. 1967; Kiyosawa 1984; Levy et al. 1991; Wang et al. 2017). In the rice/*Mo* system, at least ten such differential cultivar sets have been established, although only three, an international set, a Japanese set (JDCs) and a Chinese set (CDCs), have been widely used (Ling et al. 2004; Wu et al. 2004; Shi et al. 2015; Wang et al. 2017). As is the case for the majority of plant fungal pathogens, the race structure of *Mo* varies both temporally and spatially (Goto 1963; Bonman et al. 1986; Mekwatanakarn et al. 2000; Chen et al. 2001; Park et al. 2003; Wu et al. 2004; Consolo et al. 2008; Shi et al. 2015; Mutiga et al. 2017; Wang et al. 2017). The race structure of *Mo* populations from Cambodia (Fukuta et al. 2014), Bangladesh (Khan et al. 2016), and Japan (Kawasaki-Tanaka et al. 2016) has been identified, based on a set of differentials each harboring a single major blast resistance gene (Tsunematsu et al. 2000). However, there is no consensus to date as to the universality of this set.

Rice production in China is carried out in six distinct ecological zones, defined by topological, climatic, and edaphic parameters (Qi et al. 2006). The southern province of Guangdong (GD) belongs to Zone І, characterized by high ambient temperatures, adequate rainfall and two cropping seasons per year, and is based on the cultivation of *indica* rice cultivars. The more central province of Hunan (HN) province (Zone ІI) experiences high summer and low fall temperatures; here, rice is cropped in either one or two seasons per year, and both *indica* and *japonica* cultivars are used. In the cooler, drier northeastern provinces of Liaoning (LN) and Heilongjiang (HLJ) (Zone V), *japonica* rice cultivars are grown in just one cropping season per year. The objective of the present study was to characterize the race structure of *M*o populations collected in these four regions of the country, and at the same time to compare the utility of the CDC and JDC sets.

## Methods

### *Mo* Isolate Collection and Maintenance

Panicles exhibiting symptoms of blast disease were collected over a 3 year period, 2006–2008, from fields in each of GD, HN, LN, and HLJ provinces. The methods used to isolate single spores and to store the resulting isolates have been described elsewhere (Feng et al. 2007; Zeng et al. 2009). Each of the four *Mo* populations generated was represented by 60 monoconidial isolates; the GD population originated from 53 cultivars in 43 counties, the HN population from 51 cultivars in 15 counties, the LN population from 43 cultivars in 16 counties, and the HLJ population from 29 cultivars in 21 counties (Additional file 1: Table S1).

### Rice Differential Cultivar Sets, Inoculation and Disease Scoring

Both the CDC and JDC sets were tested for their ability to characterize the 240 *Mo* isolates. Each differential cultivar was represented by five seedlings, grown in a plastic tray (58 × 38 × 8 cm), as described by Pan et al. (2003). *Mo* inoculum was also prepared following Pan et al. (2003). Six days after inoculation, the host reaction was recorded using the Pan et al. (1996) scale, where a score of 0–2 was considered to be a resistant reaction and a score of 3–5 as susceptible. Each isolate was tested at least twice in independent inoculations, and the highest score recorded was adopted whenever there was any inconsistency.

### Race Coding

Under the CDC system, the identity of each race was defined by an alphanumeric code, which was modified from the international system (Atkins et al. 1967; All China Corporation of Research on Physiological Races of *Pyricularia oryzae* 1980; Xing et al. 2017). The letters from A to G were sequentially assigned to the seven CDCs, Tetep, Zhenlong 13, Sifeng 43, Dongnong 363, Kando 51, Hejiang 18 and Linjiangxintuanheigu (LTH), with the letter H applied to isolates which were incompatible on all seven CDCs. The races represented within a given alphabetical group were separated from one another by assigning a numerical value derived by summing the codes corresponding to the remaining differentials on which incompatible reactions produced, following by adding 1 to the numerical value (Additional file 2: Table S2). Finally the letter “Z” was inserted at the front of the designator to represent China (Zhongguo). For example, an isolate producing the reaction profile RRSRRRS (R: resistant, S: susceptible) was classified into the ZC group. From C, there were three CDCs, D, E, and F, showing resistance reactions, the race number was as 8 + 4 + 2 = 14 + 1 = 15. Thus this isolate’s race designator was given as ZC15. Under the JDC system, race designators are based on a purely numerical code (Kiyosawa 1984; Additional file 2: Table S2), where, for example, an isolate evoking the reaction pattern SSRSSRRRSSSS is given the designator 433.7 (1 + 2 + 10 + 20 + 400 + 0.1 + 0.2 + 0.4).

### Data Analysis

Six parameters were used to characterize the race structure of the four populations, namely the total race frequency *f*_*tr*_ given by (*tr/N*)*100%, the race diversity index *h*_*rdi*_ given by (*N*/*N*-1)(1-σ*x*_*i*_^*2*^), the population-specific race frequency *f*_*psr*_ given by (*psr/tr*)*100%, the population-specific race isolate frequency *f*_*psri*_ given by (*psri/N*)*100%, the dominant race isolate frequency *f*_*dri*_ given by (*dri/N*)*100%, and the top-three-dominant race isolate frequency *f*_*tdri*_ given by (*tdri/N*)*100%; in these expressions, *N* represents the number of the sum isolates of each population, *tr* the number of races identified in a given population, *x*_*i*_ the frequency of race #*i*, *psr* the number of population-specific races, *psri* the number of population-specific race isolates, *dri* the number of respective dominant race isolates, and *tdri* the number of the three predominant race isolates. A cultivar resistance gene frequency *f*_*CR*_ was calculated for each differential cultivar from the expression (*cr/N*)*100%, where *cr* represents the number of isolates which proved to be avirulent. Each cultivar was given an overall resistance rating (high: > 85%, intermediate: 60–84%, or low < 60%) based on information provided by local breeders.

## Results

### Race Diversity

According to the analysis based on the CDC set, the GD *Mo* population proved to be more diverse than any of the other three populations; its 18 races were represented in each of the alphabetical groups, producing an *f*_*tr*_ of 30.0% (Table 1 and Additional file 1: Table S1). Seven of the eight groups were detected in both the HN (17 races) and LN (12 races) populations, equivalent to *f*_*tr*_ values of, respectively, 28.3% and 20.0%. The least diverse population was from HLJ, which comprised just seven races, falling into four of the groups for an *f*_*tr*_ of 11.7%. The *h*_*rdi*_ values were quite consistent with the *f*_*tr*_ values, ranging from 0.86 (GD) to 0.50 (HLJ). The same analysis based on the JDC set was less informative; here, the four populations were concluded to comprise 30 (GD), 27 (HN), 28 (LN), and 29 (HLJ) races, but the *f*_*tr*_ values ranged from only 45.0% to 50.0%, and *h*_*rdi*_ values from 0.93 to 0.95 (Table 1).

**Table 1:** Race diversity detected by the two differential sets in the four *Mo* populations

	GD^a^	HN	LN	HLJ
CDC set^b^				
No. of race group	8	7	7	4
No. of race	18	17	12	7
Race frequency (%)	30.0	28.3	20.0	11.7
Race diversity index	0.86	0.86	0.74	0.50
JDC set				
No. of race	30	27	28	29
Race frequency (%)	50.0	45.0	46.7	48.3
Race diversity index	0.94	0.95	0.93	0.95

^a^The four populations, each consists of 60 isolates, were selected from Guangdong (GD) and Hunan (HN) in southern, and Liaoning (LN) and Heilongjing (HLJ) in northeastern parts of China (also see Additional fie 1: Table S1)

^b^CDC, Chinese differential cultivars; JDC, Japanese differential cultivars

### Specific Race Structure

The CDC set identified four population-specific races in GD, four in HN, three in LN, and just one in HLJ, generating estimates for *f*_*psr*_ of, respectively, 22.2%, 23.5%, 25.0%, and 14.3% (Tables 1 and 2). The GD-specific races were represented by five isolates, the HN-specific ones by six, the LN-specific ones by three and the HLJ-specific ones by one, giving rise to *f*_*psri*_ values of, respectively, 8.3%, 10.0%, 5.0%, and 1.7%. The JDC-based analysis predicted a greater number of population-specific races and thus generated higher estimates for both *f*_*psr*_ and *f*_*psri*_. Estimates for the former parameter were 73.3% (GD), 66.7% (HN), 67.9% (LN), and 72.4% (HLJ), and for the latter 43.3%, 65.0%, 73.3%, and 63.3%. Note that both the highest *f*_*psr*_ and the lowest *f*_*psri*_ estimates were associated with the GD population. As also concluded with respect to the race structure, the analysis based on the CDC set predicted the presence of greater diversity in the more southerly-based *Mo* populations.

**Table 2:** Specific race structures shaped by the two differential sets in the four *Mo* populations

		Race^c^		Isolate^d^	
Population^a^	Specific race (No. of isolates)^b^	No.	%	No.	%
CDC set					
GD	ZB17 (1), ZB21 (2), ZB23 (1), ZC16 (1)	4	22.2	5	8.3
HN	ZA47 (2), ZA45 (1), ZB13 (2), ZD4 (1)	4	23.5	6	10.0
LN	ZA41 (1), ZA9 (1), ZD2 (1)	3	25.0	3	5.0
HLJ	ZA59 (1)	1	14.3	1	1.7
JDC set					
GD	000.4 (1), 003.4 (1), 004.0 (2), 004.4 (2), 007.4 (2), 007.7 (1), 016.4 (1), 032.7 (1), 036.5 (1), 137.5 (1), 203.6 (1), 207.6 (1), 400.4 (1), 402.4 (1), 402.6 (1), 404.6 (1), 406.4 (1), 406.6 (1), 407.6 (1), 606.6 (1), 606.7 (1), 607.4 (1)	22	73.3	26	43.3
HN	002.0 (7), 002.3 (1), 002.6 (2), 003.0 (1), 006.0 (5), 006.6 (2), 017.7 (1), 026.4 (1), 503.2 (1), 600.4 (1), 602.2 (1), 603.2 (2), 603.6 (4), 703.0 (1), 703.2 (5), 703.6 (1), 707.4 (1), 707.6 (2)	18	66.7	39	65.0
LN	003.2 (14), 007.2 (1), 016.0 (1), 037.3 (1), 047.2 (1), 047.4 (1), 047.6 (1), 103.0 (3), 106.2 (1), 107.2 (3), 107.6 (1), 117.3 (3), 137.3 (2), 143.0 (1), 613.3 (2), 633.3 (2), 633.5 (1), 633.7 (2), 637.7 (3)	19	67.9	44	73.3
HLJ	000.1 (1), 002.4 (1), 003.7 (1), 007.5 (1), 016.5 (1) 017.5 (2), 017.3 (1), 020.4 (1), 033.1 (2), 037.7 (1), 055.7 (1), 057.5 (2), 057.7 (3), 077.1 (2), 077.7 (6), 133.1 (1), 137.3 (1), 437.2 (1), 635.5 (1), 637.5 (1), 737.1 (7)	21	72.4	38	63.3

^a^CDC, Chinese Differential Cultivar; JDC, Japanese Differential Cultivar; GD, Guangdong; HN, Hunan, LN, Liaoning, and HLJ, Heilongjing provinces of China

^b^Races were ordered by their code alphabets and numbers (also see Additional file 1: Table S1)

^c^The total of races were 18, 17, 12, 7 in GD, HN, LN, and HLJ, respectively, by CDC set, and 30, 27, 28, 29, respectively, by JDC set (also see Table 1)

^d^Each population consists of 60 isolates

### Dominant Race Structure

According to the CDC-based data, the *f*_*dri*_ values ranged widely (from 6.7% to 70.0%, see Table 3). The *f*_*tdri*_ values were 60.0% (GD), 63.3% (HN), 80.0% (LN), and 85.0% (HLJ). Races ZG1 and ZC15 were the most frequent dominant races in the two southern populations, while ZE1, ZF1, and ZA57 were the most frequent in the two northeastern ones. The JDC-based conclusions were less clear-cut. Here, *f*_*dri*_ values ranged from 8.3% to 23.3%, while the *f*_*tdri*_ values were 40.0% (GD), 43.3% (HN), 51.7% (LN), and 38.3% (HLJ) (Table 3); only one dominant race (006.4) was represented in both the GD and HN populations, and none in the LN and HLJ populations. Once again, the CDC set appeared better able to discriminate the populations’ race structure.

**Table 3:** Dominant race structures shaped by the two differential sets in the four *Mo* populations

Population^a^	Dominant races and their frequencies^b^						Top-three (%)
	1st (Isolates)	%	2nd (Isolates)	%	3rd (Isolates)	%	
CDC set							
GD	ZG1 (19)	31.7	ZC15 (10)	16.7	ZF1 (7)	11.7	60.0
HN	ZC15 (17)	28.3	ZG1 (14)	23.3	ZC13 (7)	11.7	63.3
LN	ZF1 (25)	41.7	ZE1 (18)	30.0	ZA57 (5)	8.3	80.0
HLJ	ZE1 (42)	70.0	ZA57 (5)	8.3	ZF1 (4)	6.7	85.0
JDC set							
GD	006.4 (10)	16.7	000.0 (9)	15.0	607.6 (5)	8.3	40.0
HN	006.4 (9)	15.0	002.0 (7)	11.7	006.0 (5), 703.2 (5)	16.7	43.3
LN	003.2 (14)	23.3	637.3 (5)	8.3	637.7 (3), 103.0 (3), 117.3 (3), 107.2 (3)	20.0	51.7
HLJ	037.5 (10)	16.7	737.1 (7)	11.7	077.7 (6)	10.0	38.3

^a^CDC, Chinese differential cultivar; JDC, Japanese differential cultivar; GD, Guangdong; HN, Hunan, LN, Liaoning, and HLJ, Heilongjing provinces of China

^b^Each population consists of 60 isolates

### Resistance Gene Profiling

As to both southern populations (GD vs HN), six cultivars, Tetep (98.3% vs 95.0%), Zhenlong 13 (85.0% vs 88.3%), and Kanto 51 (95.0% vs 96.7%) in the CDC set and Kusabue (90.0% vs 96.7%), Tsuyuake (93.3% vs 96.7%), and Fukunishiki (all 100%) in the JDC set, were recognized as the higher resistance cultivars (Table 4). The most deferent performances in both southern populations were specified by the three cultivars, K1 as higher resistance in GD, and intermediate resistance in HN, whereas both Dongnong 363 and K60 as intermediate resistance in GD and higher resistance in HN populations. The conclusion was that the genes *Pi1*, *Pik*, *Pik-m*, and *Piz*, along with an unknown gene(s) in Zhenlong 13, currently give adequate protection against blast disease in both provinces.

**Table 4:** Resistance frequencies of the two sets of differentials in the four *Mo* populations

		Resistance frequency (%)^c^			
Cultivar^a^	Resistance gene^b^	GD	HN	LN	HLJ
CDC set					
Tetep	*Pi1*, *Pi4*, *Pi54*	**98.3**	**95.0**	**88.3**	**90.0**
Zhenlong 13	*Pik*, *Pia*, β, ε	**85.0** ^ε^	**88.3**	**98.3**	**100**
Sifeng 43	*Pib*, *Pia*, α	68.3	41.7	**88.3** ^**α**^	**100** ^**α**^
Dongnong 363	*Pik*, *Pia*, β	76.7	**86.7**	**98.3** ^β^	**100**
Kando 51	*Pik*, γ	**95.0** ^γ^	**96.7**	51.7	15.0
Hejiang 18	*Pii*, *Pia*, δ	63.3^δ^	68.3^δ^	8.3	13.3
LTH	*Pik-l*	6.7	10.0	3.3	8.3
JDC set					
Shin 2	*Pik-s*, *Pish*, *Pi19*	63.3	58.3	6.7	11.7
Aichi Asahi	*Pia*, *Pi19*	26.7	8.3	0	11.7
Fujisaka 5	*Pii*, *Pik-s*, *Pi19*	28.3	55.0	43.3	16.7
Kusabue	*Pik*, *Pish*, *Pi19*	**90.0**	**96.7**	51.7	15.0
Tsuyuake	*Pik-m*, *Pi19*	**93.3**	**96.7**	61.7	30.0
Fukunishiki	*Piz*, *Pish*, *Pi19*	**100**	**100**	**90.0**	71.7
K1	*Pita*, *Pi19*	**98.3**	81.7	73.3	83.1
Pi4 No. 4	*Pita-2*, *Pish*	75.0	66.7	71.7	81.7
Toride 1	*Piz-t*, *Pish*, *Pi19*	68.3	65.0	71.7	80.0
K60	*Pik-p*, *Pish*, *Pi19*	80.0	**91.7**	53.3	11.7
BL1	*Pib*, *Pish*, *Pi19*	63.3	55.0	28.3	73.3
K59	*Pit*, *Pik-s*, *Pi19*	20.0	48.3	76.7	43.3

^a^CDC, Chinese differential cultivar; JDC, Japanese differential cultivar

^b^The *Pi* genes in the CDCs were adopted from Kiyosawa and Ling (1984), Mackill and Bonman (1992), Rai et al. (2011), Hua et al. (2012), and Singh et al. (2015), and those in JDCs from Kiyosawa (1981), Imbe and Matsumoto (1985); and Hayashi et al. (1998). The additional resistance genes in the CDCs given by α, β, γ, δ, and ε those were predicted in the current study by comparison of resistance frequencies between CDCs and JDCs carrying the same *Pi* genes

^c^The bold-faced ones were recognized as the higher resistance cultivar

Similar results were also identified in both northeastern populations (LN vs HLJ), four cultivars, Tetep (88.3% vs 90.0%), Sifeng 43 (98.3% vs 90.0%), Zhenlong 13 (88.3% vs 100.0%), and Dongnong 363 (98.3% vs 100.0%) in the CDC set, were considered as the higher resistance cultivars in both populations (Table 4). Note that the key *R* genes other than *Pi1* carried by such higher cultivars were denied recommending to the local breeding programs, because both *Pib* and *Pik* in LN, and *Pik* in HLJ populations were conveyed the lower resistance in the respective JDCs. Only one JDC, Fukunishiki, expressed the higher resistance in the LN population, referring the key *R* gene *Piz* as the promising one in the local breeding program. Together, the key *R* genes, *Pi1* and unknown ones in cv. Zhenlong 13 could be applied in rice breeding programs in both northeastern regions, and *Piz* and probably *Pib* (73.3% in HLJ) were as the specific ones for LN and HLJ, respectively.

## Discussion

### The CDC Set is Still Suitable to Chinese Populations of *Mo*

Both the CDC and JDC differential sets were used to explore the race structure of four Chinese *Mo* populations, allowing conclusions to be drawn regarding race diversity (Table 1), specific race structure (Table 2), and dominant race structure (Table 3). The clearest patterns were obtained from the observation of the host reaction of the members of the CDC set, where race frequency, the race diversity index, the specific race isolate frequency, and the frequency of the three predominant races were fully concordant with one another. On this basis, it was apparent that the southern and northeastern Chinese production regions support two quite distinct populations of the pathogen, with the southern populations being more diverse than the northeastern ones. The biological basis for this difference may well be that a wider range of cultivars is grown in southern as opposed to in northeastern China, which would tend to promote the diversity of the pathogen population (Wu et al. 2014; Zhang et al. 2015). The race structure predicted by the host reaction of members of the JDC set was less informative, even though this set was able to identify a greater number of races (Tables 1, 2 and 3). Following the introduction to China of the JDC set in the early 1980s, numerous attempts have been made to compare the efficacy of the two sets and to test whether their use leads to similar conclusions (Shen et al. 1986; Ling et al. 1989; Lei et al. 2000; Wu et al. 2004; Yang et al. 2004; Shi et al. 2015). The consensus has been that the JDC set is superior, largely because it is more effective in discriminating between *Mo* races (Shen et al. 1986; Ling et al. 1989; Lei et al. 2000). There might be two missing points: one is that the JDC set consists of 12 cultivars that carry different resistance genes, and the CDC set just seven cultivars that involve unknown *R* genes, which resulted in a certain difference between the sums of races identified by the two sets in a given population; and secondly, such attempts did not dissect race structures with various aspects and parameters for detailed comparison as shown in the present study. Taking consideration into the disease management and resistance cultivar breeding, the race structures established by a series of consistent, logical, and accessible parameters should be more important to rice pathologists and breeders. The coming question is whether the ideal differential set should take an ingenious balance between capacity and conformity (fitness). That would be addressed by a comprehensive comparison of several differential sets, probably including international and monogenic line sets, in more *Mo* populations.

A striking outcome of the present investigation was the identification of regionally dominant races (Table 3), which highlights the risk of potential crisis for blast outbreak in the target region (Zhu et al. 2000). This is especially the case for the northeastern region, where the combined frequency of the top three dominant isolates was > 80%.

### Some Key Resistance Genes Still Effectively Protect Chinese Rice Production

The management of disease should be bound to the utilization of genetic resistance of the host plants (He et al. 2012; Zhai et al. 2014; Deng et al. 2017; Wang et al. 2017). It is, therefore, required to have a certain and exact information on what resistance gene(s) can be used to preserve and control the regionally specific race structures (Chen et al. 2001; Wang et al. 2017; Xing et al. 2017). The differential cultivars were, in the first place, being evaluated as the useful resistance gene donors for the local breeding programs. Of the cultivars included in the CDC set, only Tetep and Zhenlong 13 showed a broad spectrum of resistance to rice blast in all four production areas (Table 4). Two further cultivars expressed a high level of resistance in three of the four populations (Dongnong 363 in HN, LN, and HLJ; Fukunishiki in GD, HN, and LN). The cultivars Kanto 51, Kusabue, and Tsuyuake, which each carry *Pik* alleles, proved to be more resistant in the southern regions than those in the northeast, while Sifeng 43 behaved in the opposite manner. Two cultivars were typed as highly resistant in just one province (K1 in GD and K60 in HN). By comparing resistance frequencies of the two differential sets that carry the same key resistance genes, the four genes, *Pi1*, *Pik*, *Pik-m*, and *Piz*, along with the as yet non-characterized genes harbored by Zhenlong 13, appear to be appropriate for rice breeding programs in either GD or HN, while *Pi1* and unknown ones in Zhenlong 13 in both northeastern rice breeding programs. The *Pita*, *Pik-p*, *Piz*, and *Pib* genes might be the specific ones for GD, HN, LN, and HLJ programs, respectively (Table 4).

As per gene-for-gene principal, resistance gene(s) identified in a given cultivar is depended on not only the resistance gene composition of the host, but also the avirulence genes involved in the isolates being tested. As to the JDCs, the first additional gene, *Pik-s*, which was carried by the Japanese susceptible cultivar Shin 2, is recognized by its interaction with the *Mo* isolate Ken Ph-03 from the Philippines (Kiyosawa 1969). The second one, *Pish*, present in seven of the JDCs, is recognized by a race isolated from the former resistance cultivar. The cultivar Reiho carries *Pita-2* (Imbe and Matsumoto 1985). The third one, *Pi19*, present in 11 of the 12 JDC entries, and is recognized by the Chinese *Mo* isolate CHNOS58–3-1 (Hayashi et al. 1998). Similarly, three additional genes, namely *Pi1*, *Pi54* (formerly *Pik-h*) borne by Tetep, and *Pik-l* by LTH, have been identified using races isolated from either China or India (Rai et al. 2011; Hua et al. 2012; Singh et al. 2015). It was noteworthy that the cultivar Tetep has been recognized as being one of the most important donors of multiple blast resistance genes (Mackill and Bonman 1992; Hua et al. 2012), while LTH is classed as a highly susceptible cultivar for the development of monogenic lines which harbors just a single blast resistance gene (Tsunematsu et al. 2000; Kawasaki-Tanaka et al. 2016). Another striking outcome of the present study is that the CDC set harbors at least five additional blast resistance genes (here assigned the temporary designations α through ε). A comparison of the resistance profiles of Sifeng 43 and BL1, which are both known to carry *Pib*, showed that the former is resistant to a greater number of the isolates from the LN and HLJ populations. This is presumed to reflect the presence of α. Similarly, the existence of the genes β, γ, and δ was inferred from the resistance profiles of, respectively, Dongnong 363 in LN, Kanto 51 in GD, and Hejiang 18 in both GD and HN (Table 4). The unidentified blast resistance genes harbored by Zhenlong 13 produced a rather similar resistance profile to that of Dongnong 363, except for isolates sampled from GD; the implication was that the blast resistance gene content of Zhenlong 13 is the same as that of Dongnong 363, with the addition of the gene ε, which conditions resistance to some GD isolates. Owning more specific and effective resistance genes in the CDC set that might be another key reason why it was more suitable for Chinese *Mo* populations, compared with the JDC set (Table 4). Similarly, the abundant resistance genes involving in the international differential cultivars, especially Raminad Str. 3, Zenith, NP125, Dular, and Sha-tiao-tsao, which was also a key determinant for its long-term use at least in America (Wang et al. 2017).

The next step forward will be to confirm such additional resistance genes through genetic approaches. This would make a certain contribution to clarify the gene compositions of the CDCs, and then provide more appropriate gene resources for the local rice breeding programs.

## Conclusions

This research has shown that the CDC set is superior to the JDC set for characterizing the race structure of Chinese *Mo* populations. Of particular concern is the potential for a serious outbreak of blast disease in the northeastern region of China, because the three most frequent races there heavily dominate the local *Mo* population. Nevertheless, the leading major blast resistance genes represented in both the CDC and JDC sets remain largely effective against *Mo* in China. The evidence from resistance profiling has suggested the presence of at least five additional blast resistance genes harbored by members of the CDC set.

## Additional Files

Additional file 1: Table S1. Samples, isolates and races of the four *Mo* populations collected in the Chinese provinces Guangdong, Hunan, Liaoning, and Heilongjiang. (DOCX 74 kb)

Additional file 2: Table S2. The membership of the Chinese and Japanese differential cultivar sets used to analyze the race structure of Chinese *Mo* populations. (DOCX 81 kb)
