# Supplementary material for: The Race Structure of the Rice Blast Pathogen Across Southern and Northeastern China
Source: Rice (N Y). 2017 Oct 5;10:46. doi: 10.1186/s12284-017-0185-y (PMC5629185; doi:10.1186/s12284-017-0185-y)
Supplement: Supplementary file 1 — Samples, isolates and races of the four Mo populations collected in the Chinese provinces Guangdong, Hunan, Liaoning, and Heilongjiang. (DOCX 74 kb) [file 12284_2017_185_MOESM1_ESM.docx]

**Table S1.** Samples, isolates, and races of the four *Mo* populations collected in Guangdong, Hunan, Liaoning, and Heilongjiang provinces, China

| **Isolate code** | **Host variety ^a^** | | | **Sample location** | **Sample time** | | **CDC race ^b^** | | **JDC race** | |
| --- | --- | --- | --- | --- | --- | --- | --- | --- | --- | --- |
| **Guangdong population** | | | |  |  | |  | |  | |
| CHL2345 | Mabayinzhan | | | Qujiang | 2008 | | ZG1 | | 006.4 | |
| CHL2357 | Shanyou 428 | | | Wengyuan | 2008 | | ZD5 | | 207.6 | |
| CHL2370 | Jinshanyou 3182 | | | Longchuan | 2008 | | ZF1 | | 406.4 | |
| CHL2375 | Shanyou 253 | | | Renhua | 2008 | | ZB31 | | 407.6 | |
| CHL2384 | Changguidao | | | Lianping | 2008 | | ZC15 | | 006.4 | |
| CHL2386 | Fengsimiao | | | Gaoyao | 2008 | | ZC16 | | 006.4 | |
| CHL2387 | Shanyou 428 | | | Meixian | 2008 | | ZC5 | | 003.4 | |
| CHL2394 | Nuo | | | Meixian | 2008 | | ZC15 | | 006.4 | |
| CHL2401 | Huayougui 99 | | | Wuhua | 2008 | | ZB17 | | 607.7 | |
| CHL2409 | Zhongerruanzhan | | | Longmen | 2008 | | ZC15 | | 006.4 | |
| CHL2412 | hanyougui 99 | | | Fengkai | 2008 | | ZG1 | | 000.0 | |
| CHL2416 | Meixiangzhan | | | Xinxing | 2008 | | ZC15 | | 006.5 | |
| CHL2417 | Tianyou 290 | | | Xingning | 2008 | | ZC15 | | 016.4 | |
| CHL2419 | Changguidao | | | Jieyang | 2008 | | ZC15 | | 007.4 | |
| CHL2421 | Sanyinian | | | Yangjiang | 2008 | | ZH | | 000.0 | |
| CHL2426 | Youyou 389 | | | Xinyi | 2008 | | ZD7 | | 607.4 | |
| CHL2433 | Huayou 86 | | | Xinyi | 2008 | | ZB23 | | 607.7 | |
| CHL2436 | Huayou 86 | | | Xinyi | 2008 | | ZB21 | | 607.6 | |
| CHL2438 | Changguidao | | | Gaozhou | 2008 | | ZC15 | | 007.4 | |
| CHL2441 | Zajiaodao | | | Gaozhou | 2008 | | ZC15 | | 036.5 | |
| CHL2442 | Tianyou 998 | | | Haifeng | 2008 | | ZF1 | | 036.4 | |
| CHL2444 | Guangyinzhan | | | Foshan | 2008 | | ZG1 | | 000.0 | |
| CHL2446 | Shuanghuangzhan | | | Jiedong | 2008 | | ZG1 | | 004.4 | |
| CHL2448 | Teyou 721 | | | Chaoan | 2008 | | ZD7 | | 406.6 | |
| CHL2449 | Zengyouzhan | | | Zengcheng | 2008 | | ZG1 | | 000.0 | |
| CHL2452 | Fengqin | | | Lianjiang | 2008 | | ZG1 | | 006.4 | |
| CHL2455 | Bairizao | | | Huidong | 2008 | | ZG1 | | 000.0 | |
| CHL2457 | Zhanyou 226 | | | Huidong | 2008 | | ZB5 | | 207.6 | |
| CHL2458 | Teyou 721 | | | Yangchun | 2008 | | ZA57 | | 002.1 | |
| CHL2464 | Taishanbai | | | Kaiping | 2008 | | ZG1 | | 004.0 | |
| CHL2467 | Meixiangzhan | | | Renhua | 2008 | | ZG1 | | 004.4 | |
| CHL2486 | Fengyou 998 | | | Lechang | 2008 | | ZH | | 000.0 | |
| CHL2490 | Guodao 1 | | | Ruyuan | 2008 | | ZB21 | | 607.6 | |
| CHL2492 | Yueyou 360 | | | Nanxiong | 2008 | | ZB7 | | 402.6 | |
| CHL2497 | Meixiangzhan | | | Qujiang | 2008 | | ZG1 | | 000.0 | |
| CHL2508 | Youyou 308 | | | Wengyuan | 2008 | | ZG1 | | 000.0 | |
| CHL2509 | Shanyou 89 | | | Pingyuan | 2008 | | ZB5 | | 007.6 | |
| CHL2513 | Shanyou 46 | | | Xinfeng | 2008 | | ZG1 | | 607.6 | |
| CHL2516 | Shanyou 82 | | | Jiaoling | 2008 | | ZF1 | | 606.6 | |
| CHL2518 | Yuxiang 88 | | | Longchuan | 2008 | | ZB5 | | 203.6 | |
| CHL2534 | Shanyou 122 | | | Meixian | 2008 | | ZD5 | | 405.6 | |
| CHL2547 | Tianyou 368 | | | Fengshun | 2008 | | ZF1 | | 607.7 | |
| CHL2549 | Shanyou 122 | | | Dapu | 2008 | | ZC15 | | 606.7 | |
| CHL2551 | Boyouyun 3 | | | Zijin | 2008 | | ZG1 | | 006.4 | |
| CHL2560 | Huanghuazhan | | | Boluo | 2008 | | ZG1 | | 006.4 | |
| CHL2561 | Longyou 665 | | | Fogang | 2008 | | ZC13 | | 006.4 | |
| CHL2563 | Zhanzinuo | | | Huidong | 2008 | | ZC15 | | 006.5 | |
| CHL2570 | Yesizhan | | | Longmen | 2008 | | ZC13 | | 007.6 | |
| CHL2581 | Qiuyou 452 | | | Qingxin | 2008 | | ZF2 | | 402.4 | |
| CHL2584 | Zayounuo | | | Huaiji | 2008 | | ZF1 | | 607.6 | |
| CHL2586 | Zhenguizhong | | | Yingde | 2008 | | ZG1 | | 006.4 | |
| CHL2593 | Qiuyou 3008 | | | Yunfu | 2008 | | ZF1 | | 607.7 | |
| CHL2600 | Qiuyou 3008 | | | Deqing | 2008 | | ZC13 | | 032.7 | |
| CHL2604 | Guinongzhan | | | Haifeng | 2008 | | ZF1 | | 007.7 | |
| CHL2606 | Xiaonongzhan | | | Zhuhai | 2008 | | ZG1 | | 000.4 | |
| CHL2608 | Taiwanyouzhan | | | Yangchun | 2008 | | ZG1 | | 000.0 | |
| CHL2615 | Bo II You 15 | | | Liangjiang | 2008 | | ZE1 | | 137.5 | |
| CHL2626 | Longyou 673 | | | Xinyi | 2008 | | ZG1 | | 400.4 | |
| CHL2631 | Shanyou 3550 | | | Gaozhou | 2008 | | ZG1 | | 004.0 | |
| CHL2636 | Bo II You 15 | | | Suixi | 2008 | | ZD5 | | 607.6 | |
| **Hunan population** | | | |  |  | |  | |  | |
| EHL0312 | | | Tefu 7 | Taoyuan | 2007 | | ZG1 | | 006.0 | |
| EHL0313 | | | Tefu 7 | Taoyuan | 2007 | | ZG1 | | 006.0 | |
| EHL0314 | | | Weiyou 402 | Anren | 2007 | | ZD7 | | 000.0 | |
| EHL0317 | | | Weiyou 402 | Anren | 2007 | | ZG1 | | 703.0 | |
| EHL0319 | | | Liangyou 287 | Liling | 2007 | | ZG1 | | 002.0 | |
| EHL0321 | | | V402 | Hengnan | 2007 | | ZH | | 000.0 | |
| EHL0323 | | | V233 | Hengdong | 2007 | | ZG1 | | 002.0 | |
| EHL0324 | | | I You 402 | Hengdong | 2007 | | ZB31 | | 002.0 | |
| EHL0326 | | | I You 402 | Hengnan | 2007 | | ZG1 | | 000.0 | |
| EHL0327 | | | Zhuliangyou 30 | Dong’an | 2007 | | ZC15 | | 002.0 | |
| EHL0329 | | | Douyuanyou 299 | Dong’an | 2007 | | ZD4 | | 703.2 | |
| EHL0330 | | | T You 99 | Dong’an | 2007 | | ZC13 | | 703.2 | |
| EHL0331 | | | Xianfeng 501 | Dong’an | 2007 | | ZC15 | | 006.4 | |
| EHL0337 | | | Jinyou 71 | Dong’an | 2007 | | ZC13 | | 006.4 | |
| EHL0338 | | | Jinyou 898 | Dong’an | 2007 | | ZC15 | | 002.0 | |
| EHL0339 | | | Nongping 001 | Dong’an | 2007 | | ZC15 | | 006.4 | |
| EHL0340 | | | Weiyou 507 | Dong’an | 2007 | | ZF2 | | 703.2 | |
| EHL0341 | | | Weiyou 298 | Dong’an | 2007 | | ZG1 | | 000.0 | |
| EHL0342 | | | Weiyou 463 | Dong’an | 2007 | | ZC5 | | 703.2 | |
| EHL0345 | | | T You 213 | Dong’an | 2007 | | ZC15 | | 006.4 | |
| EHL0346 | | | T You 103 | Dong’an | 2007 | | ZC15 | | 503.2 | |
| EHL0348 | | | Jinyou 103 | Dong’an | 2007 | | ZG1 | | 002.6 | |
| EHL0349 | | | Jinyou 402 | Dong’an | 2007 | | ZG1 | | 006.4 | |
| EHL0350 | | | Xianfeng 1 | Dong’an | 2007 | | ZC15 | | 007.6 | |
| EHL0353 | | | Nongfengyou 909 | Dong’an | 2007 | | ZC13 | | 017.7 | |
| EHL0354 | | | Jinyou 821 | Dong’an | 2007 | | ZA47 | | 703.6 | |
| EHL0355 | | | Weiyou 463 | Dong’an | 2007 | | ZG1 | | 603.6 | |
| EHL0356 | | | Zhongyou 1 | Dong’an | 2007 | | ZA47 | | 002.1 | |
| EHL0360 | | | Zhongyou 1 | Dong’an | 2007 | | ZC15 | | 002.0 | |
| EHL0361 | | | Zhuliangyou 02 | Dong’an | 2007 | | ZC15 | | 002.0 | |
| EHL0363 | | Xiangnuo | | Dong’an | 2007 | | | ZG1 | 003.0 | |
| EHL0364 | | T You 706 | | Dong’an | 2007 | | | ZF1 | 703.2 | |
| EHL0365 | | Zhongyou 493 | | Dong’an | 2007 | | | ZC13 | 006.6 | |
| EHL0367 | | Jinyou 463 | | Dong’an | 2007 | | | ZC15 | 006.6 | |
| EHL0368 | | Zhuliangyou 83 | | Dong’an | 2007 | | | ZC15 | 007.6 | |
| EHL0369 | | Jinyou 899 | | Daoxian | 2007 | | | ZB13 | 707.6 | |
| EHL0370 | | Jinyou 463 | | Daoxian | 2007 | | | ZC13 | 603.6 | |
| EHL0372 | | Nuogu | | Daoxian | 2007 | | | ZC15 | 006.0 | |
| EHL0373 | | Jinyou 463 | | Daoxian | 2007 | | | ZA45 | 002.3 | |
| EHL0374 | | Shennongdao 101 | | Daoxian | 2007 | | | ZH | 600.4 | |
| EHL0377 | | Jing 5 | | Yiyang | 2007 | | | ZC5 | 603.2 | |
| EHL0378 | | Wanxiang 13 | | Taojiang | 2007 | | | ZB5 | 603.6 | |
| EHL0379 | | Pin 3 | | Taojiang | 2007 | | | ZB31 | 607.6 | |
| EHL0380 | | Wannuo | | Yiyang | 2007 | | | ZC15 | 006.4 | |
| EHL0381 | | Wannuo | | Yiyang | 2007 | | | ZG1 | 006.0 | |
| EHL0382 | | Nuogu | | Yueyang | 2007 | | | ZB13 | 003.6 | |
| EHL0385 | | II You 58 | | Liuyang | 2007 | | | ZD5 | 707.6 | |
| EHL0386 | | Minghui 63 | | Liuyang | 2007 | | | ZG1 | 607.7 | |
| EHL0387 | | II You 838 | | Liuyang | 2007 | | | ZF2 | 707.4 | |
| EHL0393 | | Y 232 | | Youxian | 2007 | | | ZC15 | 002.6 | |
| EHL0394 | | Xinshanyou 64 | | Anren | 2007 | | | ZB7 | 603.6 | |
| EHL0395 | | Zhongyou 288 | | Huaihua | 2007 | | | ZH | 006.0 | |
| EHL0963 | | Xianghuayou 7 | | Taojiang | 2007 | | | ZC13 | 603.2 | |
| EHL0964 | | Xianghuayou 8 | | Taojiang | 2008 | | | ZB5 | 602.2 | |
| EHL0975 | | Fuyou II | | Taojiang | 2008 | | | ZC15 | 006.5 | |
| EHL0979 | | Mixiang 1 | | Changsha | 2008 | | | ZC13 | 006.4 | |
| EHL0982 | | Xiangwanxian 17 | | Changsha | 2008 | | | ZG1 | 026.4 | |
| EHL0988 | | Chaojidao 527 | | Zhuzhou | 2008 | | | ZC15 | 006.4 | |
| EHL0989 | | Xiangwanxian 13 | | Zhuzhou | 2008 | | | ZC15 | 006.4 | |
| EHL0990 | | Xiangzaoxian 24 | | Liling | 2008 | | | ZC9 | 036.4 | |
| **Liaoning population** | | | |  |  | | |  |  | |
| EHL0396 | | Minxi 12 | | Zhuanghe | 2007 | | | ZE1 | 637.1 | |
| EHL0397 | | Chenhong 59 | | Zhuanghe | 2007 | | | ZG1 | 003.2 | |
| EHL0400 | | Lige | | Zhuanghe | 2007 | | | ZE1 | 037.5 | |
| EHL0409 | | Yanjing 167 | | Donggang | 2007 | | | ZA41 | 637.7 | |
| EHL0412 | | Huanghai 6 | | Donggang | 2007 | | | ZE1 | 637.7 | |
| EHL0431 | | Liaodan 203 | | Donggang | 2007 | | | ZF1 | 107.6 | |
| EHL0433 | | Liaohan 109 | | Donggang | 2007 | | | ZF1 | 003.2 | |
| EHL0444 | | Chenhong 59 | | Pulandian | 2007 | | | ZA57 | 637.1 | |
| EHL0445 | | Minxi 12 | | Pulandian | 2007 | | | ZE1 | 003.2 | |
| EHL0446 | | Gangyu 129 | | Pulandian | 2007 | | | ZF1 | 003.2 | |
| EHL0447 | | Koshihikari | | Pulandian | 2007 | | | ZE1 | 633.5 | |
| EHL0468 | | Maoyang 3 | | Fengcheng | 2007 | | | ZF1 | 007.2 | |
| EHL0471 | | Qiao 201-2 | | Fengcheng | 2007 | | | ZE1 | 633.3 | |
| EHL0472 | | Panjin 78-2 | | Fengcheng | 2007 | | | ZE1 | 633.7 | |
| EHL0474 | | Chenhe 1 | | Fengcheng | 2007 | | | ZE1 | 633.3 | |
| EHL0484 | | Yuanfeng 9 | | Fushun | 2007 | | | ZA57 | 137.3 | |
| EHL0486 | | Fuyou 504 | | Fushun | 2007 | | | ZE1 | 016.0 | |
| EHL0489 | | Shennong 0705 | | Fushun | 2007 | | | ZH | 143.0 | |
| EHL0491 | | Jijing 88 | | Fushun | 2007 | | | ZF1 | 103.0 | |
| EHL0492 | | Xin 2 | | Fushun | 2007 | | | ZA57 | 117.3 | |
| EHL0493 | | Shennong 315 | | Fushun | 2007 | | | ZF1 | 047.4 | |
| EHL0496 | | Su 01-18 | | Dashiqiao | 2007 | | | ZF1 | 003.2 | |
| EHL0498 | | Yanfeng | | Dashiqiao | 2007 | | | ZF1 | 003.2 | |
| EHL0499 | | Yanfeng 47-12 | | Dashiqiao | 2007 | | | ZF1 | 003.2 | |
| EHL0500 | | Chenhe 1 | | Panjin | 2007 | | | ZF1 | 003.2 | |
| EHL0503 | | Maoyang 3 | | Panjin | 2007 | | | ZC9 | 633.7 | |
| EHL0505 | | Liaohe 12 | | Panjin | 2007 | | | ZF1 | 003.2 | |
| EHL0509 | | Yanjing 228 | | Panjin | 2007 | | | ZE1 | 037.3 | |
| EHL0515 | | Liaohe 1 | | Panjin | 2007 | | | ZF1 | 003.2 | |
| EHL0517 | | Huadan 995 | | Shenyang | 2007 | | | ZF1 | 003.2 | |
| EHL0519 | | Huadan 995 | | Tieling | 2007 | | | ZE1 | 613.3 | |
| EHL0879 | | Liaoxing 1 | | Dandong | 2008 | | | ZF1 | 047.2 | |
| EHL0880 | | Gangyu 2 | | Dandong | 2008 | | | ZG1 | 006.4 | |
| EHL0882 | | Chenhe 1 | | Dandong | 2008 | | | ZC15 | 006.4 | |
| EHL0883 | | Maoyang 5 | | Dandong | 2008 | | | ZE3 | 613.3 | |
| EHL0885 | | Liaojing 9 | | Dandong | 2008 | | | ZF1 | 003.2 | |
| EHL0916 | | Heixiangdao | | Dalian | 2008 | | | ZE1 | 637.3 | |
| EHL0917 | | Zhuangyan 8 | | Dalian | 2008 | | | ZE1 | 637.7 | |
| EHL0918 | | Koshihikari | | Dalian | 2008 | | | ZF1 | 003.2 | |
| EHL0919 | | Yanfeng 47 | | Dalian | 2008 | | | ZE1 | 037.1 | |
| EHL0921 | | Gangyuan 8 | | Dalian | 2008 | | | ZF1 | 107.2 | |
| EHL0922 | | Liaonong 06-7 | | Qingyuan | 2008 | | | ZC13 | 107.2 | |
| EHL0924 | | Fu 9906 | | Qingyuan | 2008 | | | ZF1 | 047.6 | |
| EHL0925 | | Fuxing 90 | | Qingyuan | 2008 | | | ZE1 | 117.3 | |
| EHL0926 | | Jijing 88 | | Qingyuan | 2008 | | | ZF1 | 106.2 | |
| EHL0927 | | Daohuaxiang | | Qingyuan | 2008 | | | ZF1 | 103.0 | |
| EHL0928 | | Liaonong 06-7 | | Xinbin | 2008 | | | ZF1 | 047.0 | |
| EHL0931 | | Jiudao 46 | | Xinbin | 2008 | | | ZF1 | 137.1 | |
| EHL0932 | | Xinyu 3 | | Xinbin | 2008 | | | ZA57 | 137.1 | |
| EHL0935 | | Jijing 88 | | Xinbin | 2008 | | | ZF1 | 103.0 | |
| EHL0936 | | Shendao 37 | | Xifeng | 2008 | | | ZC13 | 107.2 | |
| EHL0937 | | Shennong 315 | | Xifeng | 2008 | | | ZD2 | 117.3 | |
| EHL0939 | | Xinyi 3 | | Xifeng | 2008 | | | ZE1 | 077.5 | |
| EHL0940 | | Fuxing 90 | | Xifeng | 2008 | | | ZE1 | 137.3 | |
| EHL0941 | | Xinyi 3 | | Kaiyuan | 2008 | | | ZE1 | 637.3 | |
| EHL0942 | | Shennong 9903 | | Kaiyuan | 2008 | | | ZF1 | 003.2 | |
| EHL0943 | | Huadan 995 | | Kaiyuan | 2008 | | | ZA57 | 637.3 | |
| EHL0944 | | Fuxing 90 | | Kaiyuan | 2008 | | | ZA9 | 637.3 | |
| EHL0961 | | Chenhe 1 | | Suizhong | 2008 | | | ZE1 | 637.3 | |
| EHL0962 | | Yanjing 218 | | Suizhong | 2008 | | | ZC13 | 003.6 | |
| **Heilongjiang population** | | | |  |  | | |  |  | |
| EHL0524 | | Songjing 7 | | Zhaoyuan | 2006 | ZF2 | | | 002.4 | |
| EHL0527 | | Kendao 10 | | Anqing | 2006 | ZF1 | | | 003.7 | |
| EHL0532 | | Song 98-131 | | Anqing | 2006 | ZE1 | | | 077.7 | |
| EHL0540 | | Songjing 10 | | Acheng | 2006 | ZE1 | | | 077.7 | |
| EHL0547 | | Kendao 12 | | Tonghe | 2006 | ZE1 | | | 057.7 | |
| EHL0555 | | Kuyuku 131 | | Tonghe | 2006 | ZE1 | | | 037.1 | |
| EHL0557 | | Suijing 4 | | Tonghe | 2006 | ZE1 | | | 137.1 | |
| EHL0559 | | Dongnong 220 | | Shangzhi | 2006 | ZE3 | | | 020.4 | |
| EHL0560 | | Puyou 18 | | Shangzhi | 2006 | ZE1 | | | 077.7 | |
| EHL0567 | | Wuyoudao 3 | | Shangzhi | 2006 | ZA57 | | | 077.5 | |
| EHL0568 | | Wuyoudao 3 | | Shangzhi | 2006 | ZE1 | | | 077.5 | |
| EHL0570 | | Mudanjiang 19 | | Shangzhi | 2006 | ZE1 | | | 077.1 | |
| EHL0571 | | Kendao 10 | | Shangzhi | 2006 | ZE1 | | | 037.5 | |
| EHL0592 | | Shangyu 397 | | Muling | 2006 | ZE1 | | | 017.5 | |
| EHL0598 | | Punian 7 | | Hulin | 2006 | ZE1 | | | 037.5 | |
| EHL0599 | | Kenjiandao 6 | | Hulin | 2006 | ZE1 | | | 437.2 | |
| EHL0603 | | Kuyuku 131 | | Hulin | 2006 | ZE1 | | | 077.7 | |
| EHL0611 | | Kendao 12 | | Hulin | 2006 | ZE1 | | | 037.5 | |
| EHL0612 | | Kuyuku 131 | | Mishan | 2006 | ZE1 | | | 037.5 | |
| EHL0615 | | Suijing 4 | | Mishan | 2006 | ZE1 | | | 137.3 | |
| EHL0618 | | Kuyuku 131 | | Mishan | 2006 | ZH | | | 000.0 | |
| EHL0621 | | Longdun 02-318 | | Jidong | 2006 | ZF1 | | | 007.5 | |
| EHL0624 | | Kuyuku 131 | | Tangyuan | 2006 | ZH | | | 000.0 | |
| EHL0625 | | Kuyuku 131 | | Huachuan | 2006 | ZE1 | | | 037.7 | |
| EHL0629 | | Longdun 102 | | Jiamusi | 2006 | ZF1 | | | 047.0 | |
| EHL0632 | | Kuyuku 131 | | Jiamusi | 2006 | ZH | | | 000.0 | |
| EHL0635 | | Kuyuku 131 | | Baoqing | 2006 | ZE1 | | | 016.5 | |
| EHL0636 | | Kuyuku 131 | | Jixian | 2006 | ZE1 | | | 033.1 | |
| EHL0750 | | Kuyuku 131 | | Mudanjiang | 2008 | ZE1 | | | 037.5 | |
| EHL0755 | | Kuyuku 131 | | Tangyuan | 2008 | ZE1 | | | 033.1 | |
| EHL0757 | Kendao 6 | | | Tangyuan | 2008 | ZE1 | | | | 037.5 |
| EHL0764 | Kendao 11 | | | Qiang’an | 2008 | ZE1 | | | | 637.5 |
| EHL0766 | Kuyuku 131 | | | Qiang’an | 2008 | ZE1 | | | | 037.5 |
| EHL0770 | Longjing 17 | | | Qiang’an | 2008 | ZA57 | | | | 737.1 |
| EHL0776 | Kendao 6 | | | Mudanjiang | 2008 | ZE1 | | | | 635.5 |
| EHL0779 | Kuyuku 131 | | | Mudanjiang | 2008 | ZE1 | | | | 737.1 |
| EHL0781 | Longjing 17 | | | Mudanjiang | 2008 | ZA57 | | | | 737.1 |
| EHL0787 | Kenjiandao 13 | | | Jiansanjiang | 2008 | ZE1 | | | | 077.1 |
| EHL0790 | Kuyuku 131 | | | Jiangsanjiang | 2008 | ZE1 | | | | 037.5 |
| EHL0794 | Jite 639 | | | Wuchang | 2007 | ZA59 | | | | 133.1 |
| EHL0798 | Songjing 3 | | | Zhaodong | 2007 | ZE1 | | | | 077.7 |
| EHL0801 | Songjing 12 | | | Zhaodong | 2007 | ZE1 | | | | 057.7 |
| EHL0814 | Jijing 106 | | | Zhaodong | 2007 | ZE1 | | | | 057.7 |
| EHL0821 | Songjing 5 | | | Shuangcheng | 2007 | ZF1 | | | | 017.3 |
| EHL0828 | Songjing 6 | | | Shuangcheng | 2007 | ZE1 | | | | 077.7 |
| EHL0830 | Kendao 12 | | | Fangzheng | 2007 | ZE3 | | | | 055.7 |
| EHL0834 | Menggudao | | | Mudanjiang | 2007 | ZE1 | | | | 037.1 |
| EHL0835 | Menggudao | | | Mudanjiang | 2007 | ZH | | | | 000.1 |
| EHL0836 | Shangyu 397 | | | Mudanjiang | 2007 | ZE1 | | | | 637.1 |
| EHL0837 | Shangyu 397 | | | Mudanjiang | 2007 | ZE1 | | | | 037.1 |
| EHL0849 | Kuyuku 131 | | | Muling | 2007 | ZE1 | | | | 037.5 |
| EHL0850 | Kuyuku 131 | | | Muling | 2007 | ZE1 | | | | 017.5 |
| EHL0856 | Puyou 9 | | | Muling | 2007 | ZE1 | | | | 057.5 |
| EHL0858 | Puyou 9 | | | Muling | 2007 | ZE1 | | | | 057.5 |
| EHL0865 | Longjing 17 | | | Hulin | 2007 | ZE1 | | | | 737.1 |
| EHL0866 | Longjing 17 | | | Hulin | 2007 | ZE3 | | | | 037.5 |
| EHL0868 | Kenjiandao 10 | | | Hulin | 2007 | ZA57 | | | | 737.1 |
| EHL0869 | Kenjiandao 10 | | | Hulin | 2007 | ZE1 | | | | 737.1 |
| EHL0870 | Longjing 17 | | | Hulin | 2007 | ZE1 | | | | 637.3 |
| EHL0871 | Longjing 17 | | | Hulin | 2007 | ZA57 | | | | 737.1 |

^a^ The four populations, each consists of 60 isolates, were isolated and selected from 50 varieties in 43 locations in Guangdong, 51 varieties in 15 locations in Hunan, 43 varieties in 16 locations in Liaoning, and 29 varieties in 21 locations in Heilongjiang provinces in China.

^b^ CDC, Chinese differential cultivar ; JDC, Japanese differential cultivar.
