# Supplementary material for: The Race Structure of the Rice Blast Pathogen Across Southern and Northeastern China
Source: Rice (N Y). 2017 Oct 5;10:46. doi: 10.1186/s12284-017-0185-y (PMC5629185; doi:10.1186/s12284-017-0185-y)
Supplement: Supplementary file 2 — The membership of the Chinese and Japanese differential cultivar sets used to analyze the race structure of Chinese Mo populations. (DOCX 81 kb) [file 12284_2017_185_MOESM2_ESM.docx]

**Table S2.** The two sets of rice differential cultivars used in the current study for race differentiating of *Mo* isolates.

| **Differential ^a^** | **Subspecies** | **Resistance gene ^b^** | | **Race code ^c^** |
| --- | --- | --- | --- | --- |
| **CDC set** | | | | |
| Tetep | *indica* | | *Pi1*, *Pi4*, *Pi54* | A64 |
| Zhenlong 13 | *indica* | | ND | B32 |
| Sifeng 43 | *indica* | | *Pib*, *Pia* | C16 |
| Dongnong 363 | *japonica* | | *Pik*, *Pia* | D8 |
| Kando 51 | *japonica* | | *Pik* | E4 |
| Hejiang 18 | *japonica* | | *Pii*, *Pia* | F2 |
| LTH | *japonica* | | *Pik-l* | G1 |
| **JDC set** | | | | |
| Shin 2 | *japonica* | | *Pik-s*, *Pish*, *Pi19* | 1 |
| Aichi Asahi | *japonica* | | *Pia*, *Pi19* | 2 |
| Fujisaka 5 | *japonica* | | *Pii* , *Pik-s*, *Pi19* | 4 |
| Kusabue | *japonica* | | *Pik*, *Pish*, *Pi19* | 10 |
| Tsuyuake | *japonica* | | *Pik-m*, *Pi19* | 20 |
| Fukunishiki | *japonica* | | *Piz*, *Pish*, *Pi19* | 40 |
| K1 | *japonica* | | *Pita*, *Pi19* | 100 |
| Pi No. 4 | *japonica* | | *Pita-2*, *Pish* | 200 |
| Toride 1 | *japonica* | | *Piz-t*, *Pish*, *Pi19* | 400 |
| K60 | *japonica* | | *Pik-p*, *Pish*, *Pi19* | 0.1 |
| BL1 | *japonica* | | *Pib*, *Pish*, *Pi19* | 0.2 |
| K59 | *japonica* | | *Pit*, *Pik-s*, *Pi19* | 0.4 |

^a^ CDC, Chinese differential cultivar; JDC, Japanese differential cultivar.

^b^ The *Pi* genes in CDCs were adopted from Kiyosawa and Ling (1984), Mackill and Bonman (1992), Rai et al. (2011), Hua et al. (2012), and Singh et al. (2015), and those in JDCs from Kiyosawa (1981), Imbe and Matsumoto (1985), and Hayashi et al. (1998); and ND, not determined.

^c^ Race coding systems for Chinese and Japanese differentials, respectively, were adopted from The All China Corporation of Research on Physiological Races of *Pyricularia oryzae* (1980) and Kiyosawa (1984).
